# [^18^F]FDG-PET/CT in prone compared to supine position for optimal axillary staging and treatment in clinically node-positive breast cancer patients with neoadjuvant systemic therapy

**DOI:** 10.1186/s13550-021-00824-4

**Published:** 2021-08-21

**Authors:** Ariane A. van Loevezijn, Marcel P. M. Stokkel, Maarten L. Donswijk, Erik D. van Werkhoven, Marieke E. M. van der Noordaa, Frederieke H. van Duijnhoven, Marie-Jeanne T. F. D. Vrancken Peeters

**Affiliations:** 1 Department of Surgical Oncology, Netherlands Cancer Institute - Antoni Van Leeuwenhoek, Plesmanlaan 121, 1066 CX Amsterdam, The Netherlands; 2 Department of Nuclear Medicine, Netherlands Cancer Institute - Antoni Van Leeuwenhoek, Plesmanlaan 121, 1066 CX Amsterdam, The Netherlands; 3 Department of Biometrics, Netherlands Cancer Institute - Antoni Van Leeuwenhoek, Plesmanlaan 121, 1066 CX Amsterdam, The Netherlands; 4 Department of Surgery, Amsterdam University Medical Center, Meibergdreef 9, 1105 AZ Amsterdam, The Netherlands

**Keywords:** FDG-PET/CT, Breast cancer, Neoadjuvant chemotherapy, Axillary staging, Prone imaging

## Abstract

**Purpose:**

Axillary staging before neoadjuvant systemic therapy in clinically node-positive breast cancer patients with tailored axillary treatment according to the Marking Axillary lymph nodes with radioactive iodine seeds (MARI)-protocol, a protocol developed at the Netherlands Cancer Institute, is performed with [^18^F] fluorodeoxyglucose (FDG) positron emission tomography and computed tomography (PET/CT). We aimed to assess the value of FDG-PET/CT in prone compared to standard supine position for axillary staging.

**Methods:**

We selected patients with FDG-PET/CT in supine and prone position who underwent the MARI-protocol. One hour after administration of 3.5 MBq/kg, [^18^F]FDG-PET was performed with a low-dose prone position CT-thorax followed by a supine whole-body scan. Scans were separately reviewed by two nuclear medicine physicians and categorized by number of FDG-positive axillary lymph nodes (ALNs; cALN<4 or cALN≥4). Main outcome was axillary up- or downstaging.

**Results:**

Of 153 patients included, 24 (16%) patients were up- or downstaged at evaluation of prone images: One observer upstaged 14 patients, downstaged 3  patients and reported a higher number of ALNs (3.6 vs. 3.2, *p* < 0.001), while staging (4 up- and 5 downstaged) and number of ALNs (2.8 vs. 2.8) did not differ for the other. Observers agreed on up- or downstaging in only 1 (1%) patient. Irrespective of supine or prone position scanning, observers agreed on axillary staging in 124 (81%) patients and disagreed in 5 (3%). Interobserver agreement was lower with prone assessments (86%, *K* = 0.67) than supine (92%, *K* = 0.80).

**Conclusions:**

Axillary staging with FDG-PET/CT in prone compared to supine position did not result in concordant up- or downstaging. Therefore, FDG-PET/CT in supine position only can be considered sufficient for axillary staging.

**Supplementary Information:**

The online version contains supplementary material available at 10.1186/s13550-021-00824-4.

## Introduction

Treatment of clinically node-positive (cN+) breast cancer patients increasingly consists of neoadjuvant systemic therapy (NST), allowing for response monitoring and potential local–regional treatment de-escalation [1]. Patients with a pathological complete response (pCR) of the axilla after NST have improved prognosis compared to patients with residual axillary disease [2] and are unlikely to benefit from axillary lymph node dissection (ALND). The optimal axillary treatment strategy for cN+ patients treated with NST, however, is yet unknown.

Several studies are currently investigating de-escalation of axillary treatment in cN+ patients with excellent response to NST [3–12]. At the Netherlands Cancer institute (NKI), the Marking Axillary lymph nodes with Radioactive Iodine seeds (MARI)-protocol was developed [7, 8, 11, 13]. The MARI-protocol selects patients for response-adjusted axillary treatment based on the presence of less or more than four (cALN<4 or cALN≥4) positive axillary lymph nodes on pre-NST acquired [^18^F]fluorodeoxyglucose (FDG) positron emission tomography and computed tomography (PET/CT) in combination with the MARI-procedure, in which the largest tumor-positive axillary lymph node is marked with an ^125^iodine seed pre-NST (MARI-node) and selectively removed and assessed post-NST [7, 8, 11].

FGD-PET/CT is not considered a standard diagnostic modality for breast cancer staging in most institutions. Current Dutch guidelines recommend staging with FDG-PET/CT in all patients with clinical stage≥T3 and/or N+ breast cancer [14]. The use of FDG-PET/CT in cN+ breast cancer patients provides improved axillary and regional staging compared to other imaging modalities [15–17] and is typically performed with a whole-body scan in supine position. At the NKI, a prone position scan (with hanging breasts) is added to the standard supine position whole-body FDG-PET/CT scan. The prone position scans are used for quantification of uptake in the primary tumor with a higher reproducibility compared to the supine PET/CT scans [18–26]. Some studies suggest that prone position FDG-PET/CT also improves axillary staging [18, 27, 28].

Because the MARI-protocol relies on the distinction between less than four (cALN<4) and more than four (cALN≥4) positive axillary nodes, performing axillary staging with FDG-PET/CT either in prone or supine position could affect treatment according to the MARI-protocol. In this study, we therefore aimed to assess the clinical value of FDG-PET/CT in prone compared to supine position for axillary staging in cN+ patients treated according to the MARI-protocol.

## Methods

### Patient selection

This retrospective cohort study included 159 women who underwent response-adjusted axillary treatment according to the MARI-protocol, of whom data have been previously published [29]. Patients with a pre-NST acquired [^18^F]FDG-PET/CT in supine and prone position of 18 years or older with stage II–III pathologically proven cN+ breast cancer of any subtype that underwent the MARI-procedure at the NKI between July 2014 and September 2017 were included. Exclusion criteria were history of breast cancer or a non-FDG-avid breast cancer. This study was approved by the institutional review board of the NKI.

### FDG-PET/CT acquisition

**Fig. 1:**
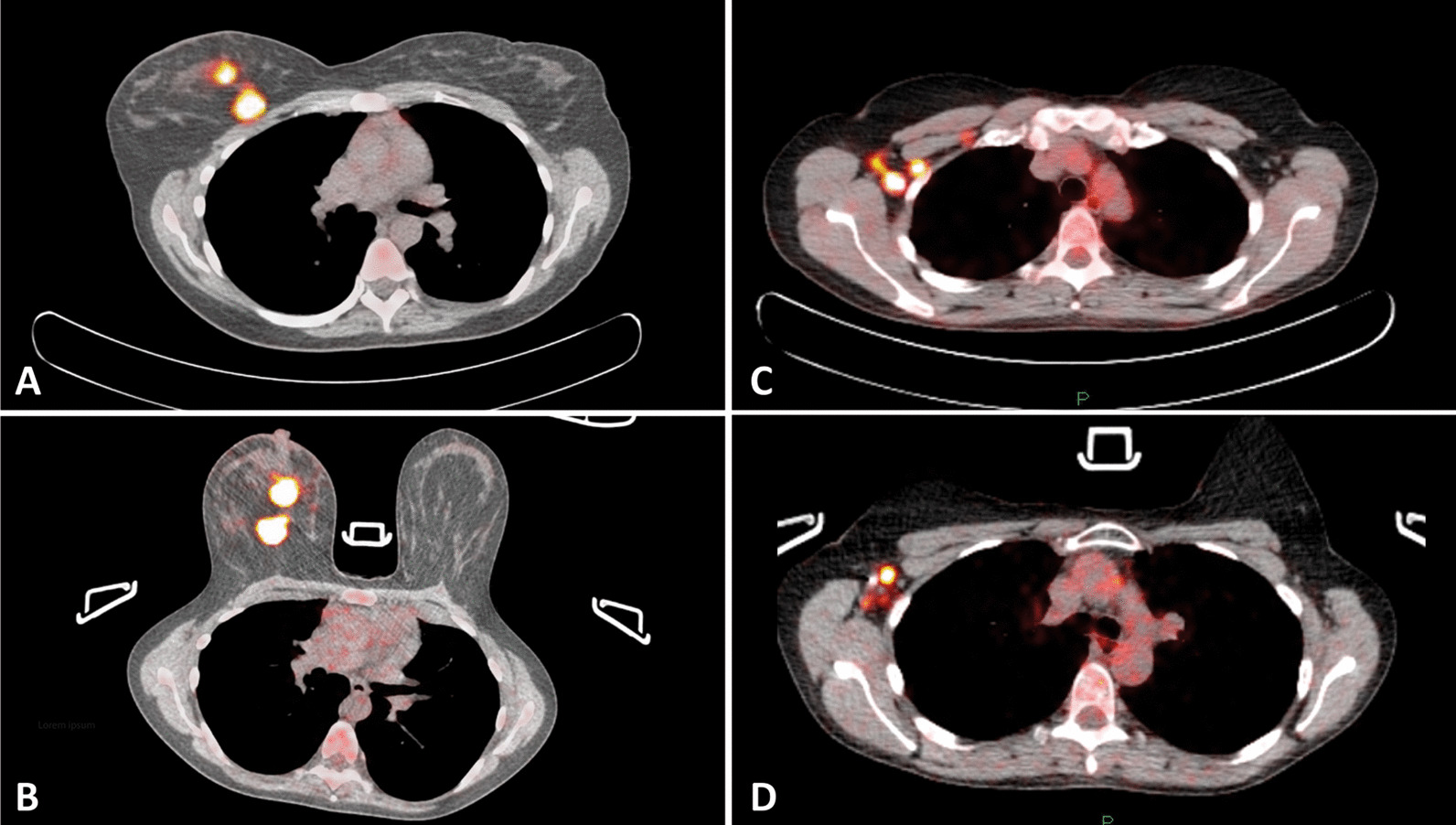
Patient with multifocal right-sided breast cancer with [^18^F]FDG-PET/CT performed in supine and prone position. Images **A** and **C** were acquired in supine position and images **B** and **D** were acquired in prone position, showing a multifocal FDG-avid primary breast cancer and multiple FDG-positive axillary lymph node metastases, respectively. In this patient, one additional FDG-positive ALN was counted in prone position compared to supine position

Patients fasted for ≥ 6 h and received oral prehydration before intravenous injection of 120–400 MBq FDG according to their weight (3.5 MBq/kg). After a resting period of 60 ± 10 min, PET/CT studies were acquired using a whole-body PET/CT scanner (Gemini TF, Philips, Cleveland, OH). First, a non-contrast-enhanced low-dose CT (ldCT) scan (dose modulated, 40mAs, 2 mm slice thickness) of the thorax was performed in prone position using a mock-up coil for hanging breast imaging (Fig. 1), followed by PET acquisition (3 min per bed position, 2 mm voxel reconstruction). Subsequently, a whole-body PET (1.5 min per bed position, 4 mm voxel reconstruction) combined with ldCT scan was performed in supine position. The ldCTs were used for attenuation correction and anatomical localization. Prone and supine scans were both performed with the arms of the patient placed in identical positions above the head, to facilitate optimal imaging of the axillary nodes.

### Image reading

For purpose of this study, [^18^F]FDG-PET/CT images were retrospectively reviewed by two experienced nuclear medicine physicians with an interval of more than seven days between respective assessments of prone and supine images. The observers were blinded to clinical and other imaging results. Both observers separately assessed the total number of FDG-positive level I and II axillary lymph nodes. A lymph node was regarded as FDG-positive when the uptake was higher than the non-specific blood pool activity.

Clinical staging of the axilla at time of treatment was performed by experienced nuclear medicine physicians who assessed both prone and supine position FDG-PET/CT images. The total number of FDG-positive ALNs identified was reported. All PET/CT scans were discussed during multidisciplinary consultation prior to surgery in order to confirm correct axillary staging and treatment strategy. According to the MARI-protocol, the number of FDG-positive axillary nodes was used to stage the axilla rather than the clinical TNM classification, in which the N-classification also refers to internal mammary and peri-clavicular nodes. Patients with less than four FDG-positive axillary nodes on PET/CT were categorized as cALN<4 and patients with four or more FDG-positive nodes were categorized as cALN≥4.

### Tailored axillary treatment according to the MARI-protocol

A comprehensive description of the MARI-procedure and radiation safety protocols has been described previously [13]. In summary, the largest pathology proven tumor-positive axillary node (i.e., MARI-node) is marked with an ^125^iodine seed under ultrasound guidance, prior to the first NST cycle. The iodine seeds used for localization of the MARI-node have an apparent activity varying from 0.2 to 1.0 MBq at time of implementation, which is lower than that for breast tumor localization (1.0–7.6 Mbq) [30, 31]. Localization and marking of breast tumor(s) were performed during the same procedure. Marking of the axilla and breast was followed by ultrasound and/or mammography to confirm adequate position of the marker(s).

After completion of NST, a gamma probe was used to localize the Iodine seed(s) and guide surgical resection. Excision of the MARI-node was performed simultaneously with surgery of the breast. An intraoperative frozen section of the MARI-node was performed in all cALN≥4 patients.

Pathological complete response (pCR) of the axilla (ypN0) was defined as the absence of vital tumor cells in all removed ALNs, irrespective of the response in the breast. All cALN<4 patients with pCR of the MARI-node (ypMARI-neg) received no further axillary treatment. Patients cALN<4 without pCR of the MARI-node (ypMARI-pos) and cALN≥4 patients with pCR of the MARI-node (ypMARI-neg) received axillary radiotherapy (levels I to IV). Patients staged cALN≥4 with residual tumor cells in the MARI-node (ypMARI-pos) received both ALND and ART.

### Outcomes

The primary outcome was the number of patients who were upstaged or downstaged with prone position FDG-PET/CT compared to supine FDG-PET/CT. Patients could either be upstaged to cALN≥4 or downstaged to cALN<4. Secondary outcomes were differences in the number of ALNs counted as FDG-positive and interobserver agreement, assessed as the agreement on axillary staging category between the two nuclear medicine physicians on both prone and supine FDG-PET/CT images. The original axillary staging report containing both prone and supine FDG-PET/CT scan images was used as a reference standard. In addition, three-year axillary recurrence-free interval was assessed by axillary treatment and compared with observer agreement.

### Statistical analysis

Differences in axillary staging category between prone and supine position observer assessments were calculated with McNemar's test. Differences in the reported number of ALNs were calculated using Wilcoxon signed-rank test. The agreement between the two observers was evaluated using Cohen’s kappa, and of the observers and the original report using Fleiss’ kappa (separately for observations in supine and prone position). Recurrence-free survival was defined as the time from the MARI-procedure to recurrence or death by any cause. The Kaplan–Meier method was used to estimate recurrence-free survival rates. All survival estimates were reported with their 95% confidence intervals. The two-sided 95% confidence intervals for proportions were calculated using the Clopper–Pearson exact method. Statistical significance for comparisons between groups was defined as *p* < 0.05. All statistical analyses were performed in IBM SPSS Statistics, version 25.0.

## Results

### Patient and treatment characteristics

Six of the 159 patients analyzed were excluded due to missing prone position FDG-PET/CT, leaving 153 patients for analysis. Baseline patient characteristics are shown in Table 1. Median age was 49 years (range 22–79). The majority of patients had invasive ductal carcinoma (89%) and HR-positive/HER2-negative tumor subtype (46%). The mean number of suspect nodes identified according to axillary staging at time of treatment with combined supine and prone FDG-PET/CT images was 3.2 (range 1–14). The number of FDG-positive ALNs was cALN<4 in 108 (71%) patients and cALN≥4 in 45 (29%) patients.

**Table 1:** Baseline patient characteristics (*N* = 153)

Age, (y)	49	(39–55)
Diagnostic imaging		
Tumor size MRI (mm)	30	(22–47)
FDG-positive ALNs	2	(1–4)*
Clinical tumor stage		
cT ≤ 1	33	(22%)
cT2	86	(56%)
cT ≥ 3	33	(22%)
Clinical axillary stage		
cALN<4	108	(71%)
cALN≥4	45	(29%)
Histology		
Ductal	136	(89%)
Lobular	15	(10%)
Other	2	(1%)
Tumor subtype		
HR+/HER2−	71	(46%)
HR+/HER2+	24	(16%)
HR−/HER2+	18	(12%)
Triple-negative	40	(26%)
Grade		
Grade 1	3	(2%)
Grade 2	77	(53%)
Grade 3	65	(45%)
Unknown	7	–

Data are median (IQR) or *N* (%)

*The number of FDG-avid nodes was reported as ≥ 10 in 15 patients, in which case the cut-off value of 10 was used

*cALN*<*4* less than four FDG-positive axillary lymph nodes, *cALN*≥*4* more than four FDG-positive axillary lymph nodes, *MARI* marking axillary lymph nodes with radioactive iodine seeds, *ALNs* axillary lymph nodes

**Fig. 2:**
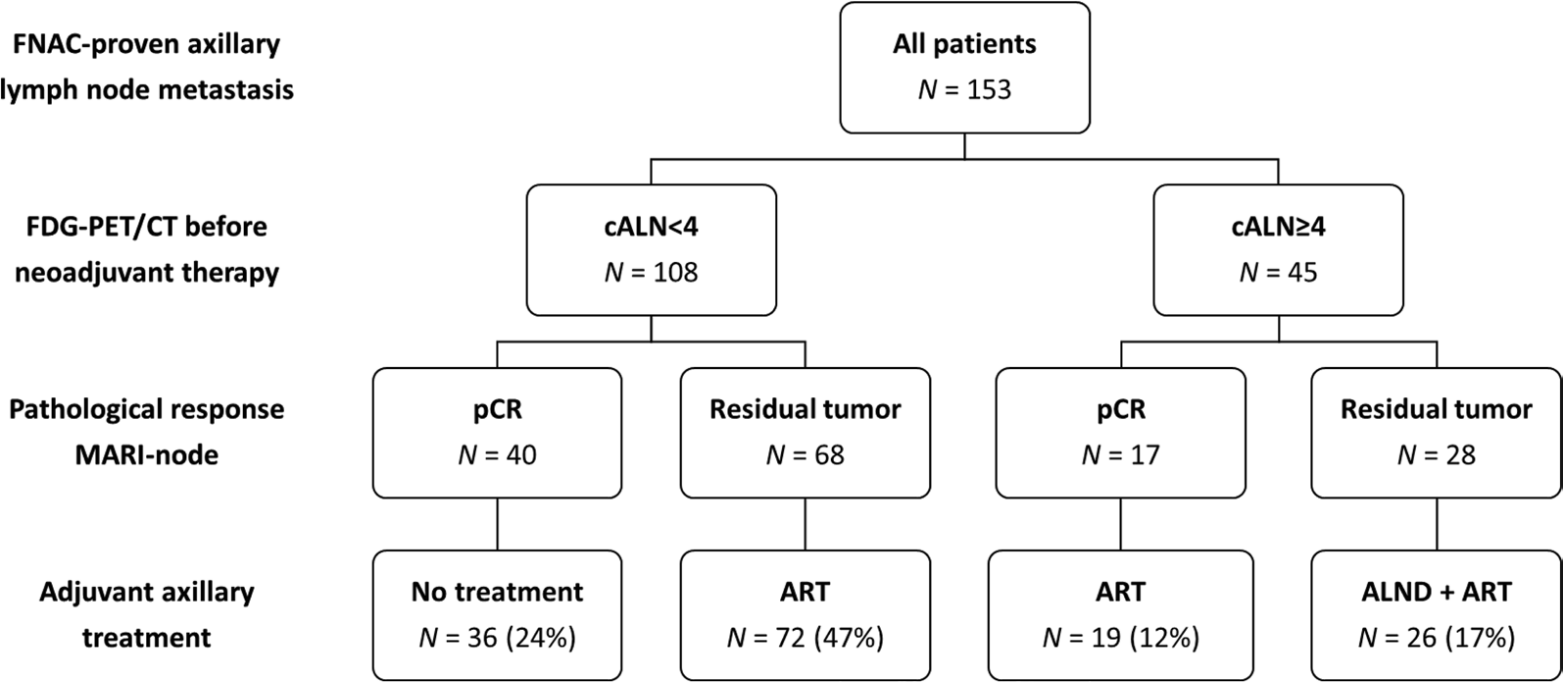
Tailored axillary treatment according to the MARI-protocol. Protocol deviations occurred in 10 patients: six cALN<4 patients with pCR of the MARI-node received ART, two cALN<4 patients without MARI-node pCR received no further treatment and two cALN≥4 patients without MARI-node pCR did not undergo ALND. *FNAC* fine-needle aspiration cytology, *cALN*<*4* less than four FDG-positive axillary lymph nodes, *cALN*≥*4* more than four FDG-positive axillary lymph nodes, *pCR* pathological complete response*, ART* axillary radiotherapy, *ALND* axillary lymph node dissection

After NST, pCR of the MARI-node was found in 57 (37%; 95% CI 30–45) patients. Breast pCR occurred in 42 (27%; 95% CI 21–35) patients and 39 (25%; 95% CI 19–33) patients had pCR of the breast and MARI-node (ypT0N0). Response-adjusted axillary treatment according to the MARI-protocol resulted in 36 (24%) patients receiving no further axillary treatment, 91 (59%) patients underwent ART (72 cALN<4, ypMARI-pos and 19 cALN≥4, ypMARI-neg) and 26 (17%) patients underwent ALND plus ART (Fig. 2).

### Clinical value of prone FDG-PET/CT for axillary staging

**Fig. 3:**
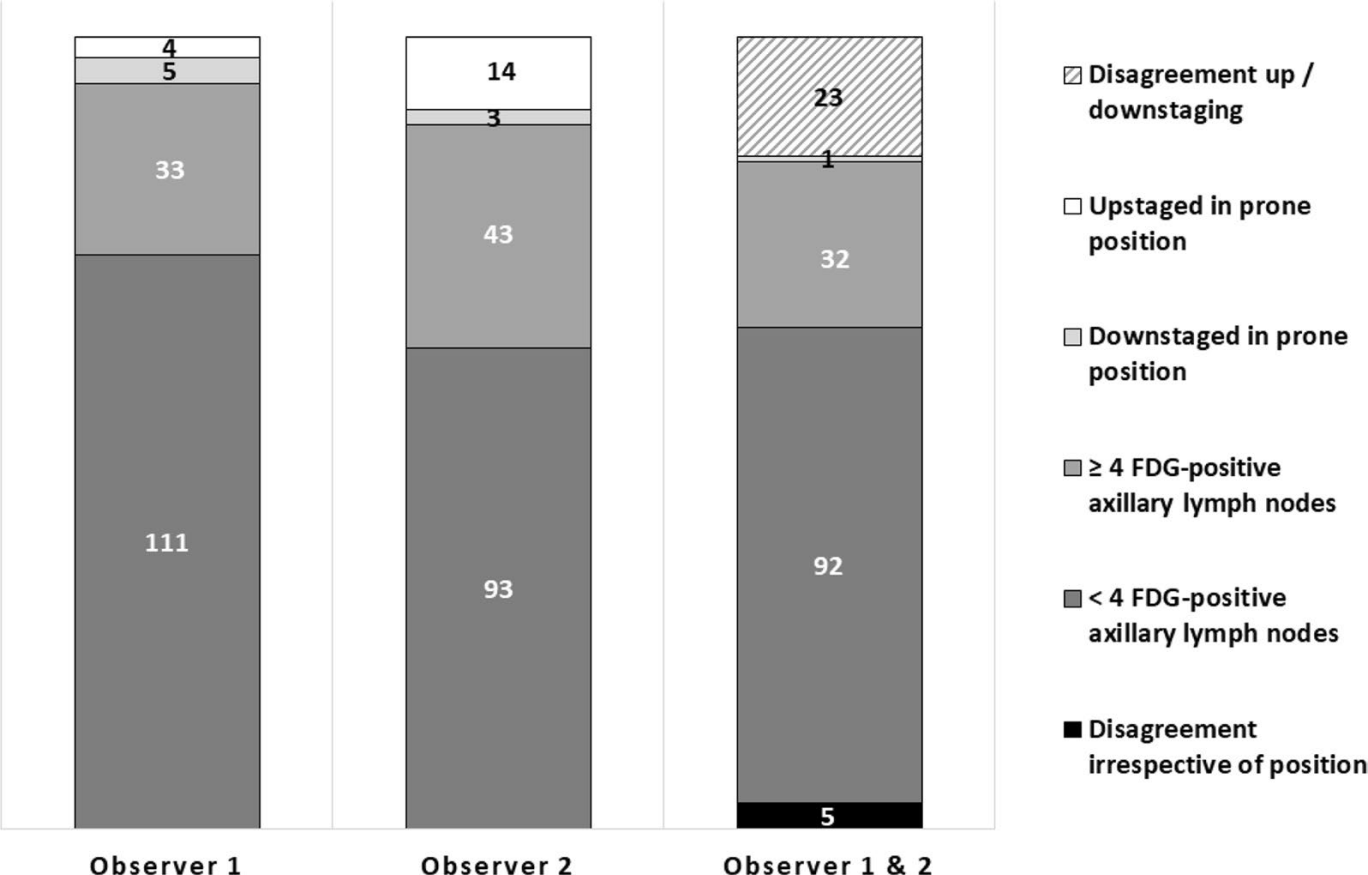
Up- and downstaging of the axilla with [^18^F]FDG-PET/CT in prone compared to supine position. Observers disagreed on up-/downstaging with prone FDG-PET/CT in 23 patients and agreed 1 patient, who was downstaged. Observers agreed on staging irrespective of scan position in 124 patients: 32 had more than four (stage cALN≥4) FDG-avid axillary lymph nodes and 92 had less than four (stage cALN<4) FDG-avid axillary nodes. Observers disagreed irrespective of scan position in 5 patients

Down- and upstaging with prone position compared to supine position FDG-PET/CT were evaluated for the two study observers. Irrespective of the patients scan position (i.e., prone or supine), both observers agreed on axillary staging in 124 (81%; 95% CI 74–87) patients and disagreed in 5 (3%; 95% CI 1–7). These five patients were consistently staged cALN<4 by the first observer and cALN≥4 by the second observer (Fig. 3, Table S1 [online resource 1]). In total, twenty-four (16%; 95% CI 10–22) patients were up- or downstaged at assessment of prone images by either one of the observers. Two of these patients were counted by both observers: Observers agreed on up- or downstaging with prone FDG-PET/CT in only one patient (1%; 95% CI 0–4), who was downstaged by both observers, and one patient was downstaged by observer 1 while upstaged by observer 2.

The first observer upstaged 4 patients, downstaged 5 patients and did not report a higher mean number of FDG-positive ALNs (2.8 vs. 2.8, *p* = 0.358). The second observer upstaged 14 patients and downstaged 3 patients and did report a higher mean number of FDG-positive ALNs (3.6 vs. 3.2, *p* < 0.001).

Axillary staging changed at assessment of prone scan images for the first observer in a total of 9 (6%; 95% CI 3–11) patients and for the second observer in 17 (11%; 95% CI 7–17) patients (Fig. 2). For the first observer, this change was in concordance with the original multidisciplinary determined staging category in 4 patients (3 upstaged, 1 downstaged) and in discordance in 5 patients (1 upstaged, 4 downstaged). For the second observer, the changed category was in concordance with the original report in 9 patients (6 upstaged, 3 downstaged) and in discordance in 8 patients (all upstaged).

### Interobserver agreement

Interobserver agreement on axillary staging was greater with FDG-PET/CT assessments in supine compared to prone position (*K* = 0.80 vs. *K* = 0.67) (Table 2). At assessment of supine position FDG-PET/CTs, a total of 141 (92%) patients were categorized in the same axillary staging group (cALN<4 or cALN≥4) and 131 (86%) patients were categorized in the same axillary staging group at assessment of prone position scans. Observer agreement was also greater using FDG-PET/CT in supine compared to prone position only when observations were compared to the original staging category based on both prone and supine FDG-PET/CT scan images together (Fleiss *K* = 0.78 vs. 0.74) (Table 2).

**Table 2:** Interobserver agreement on axillary staging with FDG-PET/CT in supine or prone position

	Axillary staging						Agreement					
	Observer 1		Observer 2		Original report		Observers		Kappa	Obs. & report		*Kappa
Supine PET/CT									0.80			0.78
cALN<4	115	75%	107	70%	108	71%	105	69%		99	65%	
cALN≥4	38	25%	46	30%	45	29%	36	24%		34	22%	
Total	153	100%	153	100%	153	100%	141	92%		133	87%	
Prone PET/CT									0.67			0.74
cALN<4	116	76%	96	63%	108	71%	95	62%		93	61%	
cALN≥4	37	24%	57	37%	45	29%	36	24%		35	23%	
Total	153	100%	153	100%	153	100%	131	86%		128	84%	

Data are *N*, %.*Fleiss kappa. *Original report* staging at time of treatment including both supine and prone FDG-PET/CT scans

*cALN*<*4* less than four FDG-positive axillary lymph nodes, *cALN*≥*4* more than four FDG-positive axillary lymph nodes

### Axillary recurrences

Median follow-up was 3.8 years (interquartile range [IQR] 2.9–4.6). Axillary recurrences occurred in four (3%) patients, all with synchronous other metastases, resulting in a three-year axillary recurrence-free interval of 98% (95% CI 96–100). At review of FDG-PET/CT images acquired in prone position, one of the study observers disagreed with the originally assigned staging category in one of these four patients with axillary metastases and upstaged the patient cALN≥4, which would have resulted in ART instead of no further treatment.

## Discussion

In this study, we found that axillary staging in cN+ patients undergoing tailored axillary treatment according to the MARI-protocol using prone position FDG-PET/CT did not result in substantial up- or downstaging compared to axillary staging with FDG-PET/CT in standard supine position. Although one of the two observers reported a higher mean number of FDG-positive ALNs (3.6 vs. 3.2) with FDG-PET/CT in prone position, the other observer did not (2.8 vs. 2.8). Moreover, observers agreed on up- or downstaging with prone position FDG-PET/CT in only one (1%) out of 153 patients, who was downstaged.

Accurate axillary staging prior to NST is essential when aiming to de-escalate treatment after NST, which is now increasingly being proposed for cN+ patients [3–8, 10, 12, 20]. The gold standard of ALND is being replaced by more response-adjusted strategies, such as the MARI-protocol or the post-NST selective assessment of a clipped node and a sentinel-node (e.g., targeted axillary dissection) [3, 10–12].

FDG-PET/CT is currently the most accurate imaging modality to stage the axilla in cN+ patients and is standard performed with the patient in supine position [15–17]. More recently, several studies found that breast cancer staging with FDG-PET/CT in prone position improves visualization and diagnosis of small, deep-seated lesions in the breast and close to the chest wall at higher standardized uptake values (SUVs), due the separation of deep breast structures and relaxation of the pectoralis muscle [18–26]. The difference in image reading between the scan positions concerns mainly the decompression of soft breast tissue. Therefore, scanning in prone position is less likely to be advantageous when assessing the firmer structures of the axilla. In addition to the evaluation of the breast tumor, MRI is considered the most accurate staging method [32].

Breast cancer staging with FDG-PET/CT in supine position has a high accuracy and a relatively good interobserver agreement when classifying lesions in the breast [23, 24, 33, 34]. At present, only few studies addressed interobserver agreement on axillary staging with supine FDG-PET/CT [23, 27]. To our knowledge, interobserver agreement has not been reported for scanning in prone position. We found excellent observer agreement on axillary staging with supine position imaging (*K* = 0.80) but less agreement with prone position imaging (*K* = 0.67). A greater observer agreement with assessments of FDG-PET/CT in supine compared to prone position was also found when results were compared to the original axillary staging report (Fleiss *K* = 0.78 vs. 0.73).

Two studies have compared the accuracy of FDG-PET/CT in supine and prone position scanning for axillary staging in cN+ patients [18, 27]. In the study by Abramson et al. [27], three scan observers identified an equal numbers of involved lymph nodes on prone and supine scanning in 12 out of 16 patients and in 4 patients, prone scanning resulted in a higher number of visualized lymph nodes. Interobserver discrepancies were resolved at a consensus reading. However, as in our current study, pathology analysis of the ALNs was not performed because patients underwent NST prior to surgery.

Teixeira et al. [18] compared prone and supine position FDG-PET/CT for the visualization of primary tumors and regional lymph node metastases in a cohort of 198 stage II/III breast cancer patients. In this study, a slightly higher number of ALNs was reported with prone versus imaging (IQR 2–5 vs. 1–4). No differences were found for the detection of extra-axillary nodes. This study also reported higher maximum SUVs for the primary tumor, as well as the lymph nodes with prone position imaging compared to supine. Interobserver variability was not addressed, and confirmative pathology analysis of the ALNs had not been performed.

The detection of a slightly higher number of FDG-positive ALNs with prone position FDG-PET/CT may not affect axillary staging as defined by the MARI-protocol. In the present study, observers did not agree on upstaging with prone position FDG-PET/CT in any of the patients. Furthermore, an equally high three-year axillary RFS of 98% was recently reported in all patients who underwent the MARI-protocol at the Netherlands Cancer Institute, which included more than one-third of patients who were staged using FDG-PET/CT in supine position only [35].

Limitations of this study are that we could not compare prone and supine position axillary staging with the gold standard of ALND, because all patients underwent NST and received response-adjusted axillary treatment. Instead, we used the original axillary staging report as the reference standard, because it included both supine and prone FDG-PET/CT images and was discussed at multidisciplinary consultations.

Notably, we recommend consensus readings if the interpretation of FDG-PET/CT images affects the proposed axillary treatment. At the Netherlands Cancer Institute, all patients scheduled for tailored axillary treatment according to the MARI-protocol are discussed at multidisciplinary consultations, where all available FDG-PET/CT scans, corresponding diagnostics and the proposed axillary treatment are evaluated. This could further diminish the reported interobserver variability of 8% for standard supine and 14% for prone position FDG-PET/CT axillary staging.

In conclusion, re-evaluation of the number of FDG-positive ALNs on FDG-PET/CT in prone compared to standard supine position scanning did not concordantly change axillary staging in cN+ breast cancer patients. Moreover, observer agreement was greater with FDG-PET/CT in supine position only. Therefore, we found no added value of scanning in prone position for axillary staging in cN+ breast cancer patients with tailored axillary treatment after NST according to the MARI-protocol.

## Supplementary Information

**Additional file 1.Table S1.** Number of patients categorized in the same axillary staging groups with FDG-PET/CT in supine and prone position by both observers.

## Data Availability

The datasets generated for the current study are available from the corresponding author on reasonable request.

## Abbreviations

ALNs: Axillary lymph nodes
ALND: Axillary lymph node dissection
ART: Axillary radiotherapy
cALN<4: Less than four FDG-positive axillary lymph nodes
cALN≥4: More than four FDG-positive axillary lymph nodes
cN+: Clinically node-positive
FDG: Fluorodeoxyglucose
FNAC: Fine-needle aspiration cytology
MARI: Marking axillary lymph nodes with radioactive iodine seeds
MARI-node: The largest tumor-positive axillary lymph node marked with an ^125^iodine seed
NST: Neoadjuvant systemic therapy
NKI: Netherlands Cancer institute
PET/CT: Positron emission tomography and computed tomography
pCR: Pathological complete response
RFS: Recurrence-free survival
ypMARI-neg: Pathology analysis of the MARI-node after NST is tumor-negative
ypMARI-pos: Pathology analysis of the MARI-node after NST is tumor-positive

## References

[1] Jones RL, Smith IE (2006). Neoadjuvant treatment for early-stage breast cancer: opportunities to assess tumour response. Lancet Oncol.

[2] Fayanju OM, Ren Y, Thomas SM, Greenup RA, Plichta JK, Rosenberger LH (2018). The clinical significance of breast-only and node-only pathologic complete response (pCR) after neoadjuvant chemotherapy (NACT): a review of 20,000 breast cancer patients in the National Cancer Data Base (NCDB). Ann Surg.

[3] Caudle AS, Yang WT, Krishnamurthy S, Mittendorf EA, Black DM, Gilcrease MZ (2015). Improved axillary evaluation following neoadjuvant therapy for patients with node-positive breast cancer using selective evaluation of clipped nodes: implementation of targeted axillary dissection. J Clin Oncol.

[4] Boughey JC, Suman VJ, Mittendorf EA, Ahrendt GM, Wilke LG, Taback B (2013). Sentinel lymph node surgery after neoadjuvant chemotherapy in patients with node-positive breast cancer: the ACOSOG Z1071 (Alliance) clinical trial. JAMA.

[5] Boileau JF, Poirier B, Basik M, Holloway CM, Gaboury L, Sideris L (2015). Sentinel node biopsy after neoadjuvant chemotherapy in biopsy-proven node-positive breast cancer: the SN FNAC study. J Clin Oncol.

[6] Choi HJ, Kim I, Alsharif E, Park S, Kim JM, Ryu JM (2018). Use of Sentinel lymph node biopsy after neoadjuvant chemotherapy in patients with axillary node-positive breast cancer in diagnosis. J Breast Cancer.

[7] Donker M, Straver ME, Wesseling J, Loo CE, Schot M, Drukker CA (2015). Marking axillary lymph nodes with radioactive iodine seeds for axillary staging after neoadjuvant systemic treatment in breast cancer patients: the MARI procedure. Ann of Surg.

[8] Koolen BB, Donker M, Straver ME, van der Noordaa MEM, Rutgers EJT, Valdes Olmos RA (2017). Combined PET-CT and axillary lymph node marking with radioactive iodine seeds (MARI procedure) for tailored axillary treatment in node-positive breast cancer after neoadjuvant therapy. Br J Surg.

[9] Pilewskie M, Morrow M (2017). Axillary nodal management following neoadjuvant chemotherapy: a review. JAMA oncol.

[10] Simons JM, van Nijnatten TJA, van der Pol CC, Luiten EJT, Koppert LB, Smidt ML (2019). Diagnostic accuracy of different surgical procedures for axillary staging after neoadjuvant systemic therapy in node-positive breast cancer: a systematic review and meta-analysis. Ann Surg.

[11] van der Noordaa MEM, van Duijnhoven FH, Straver ME, Groen EJ, Stokkel M, Loo CE (2018). Major reduction in axillary lymph node dissections after neoadjuvant systemic therapy for node-positive breast cancer by combining PET/CT and the MARI procedure. Ann Surg Oncol.

[12] van Nijnatten TJA, Simons JM, Smidt ML, van der Pol CC, van Diest PJ, Jager A (2017). A novel less-invasive approach for axillary staging after neoadjuvant chemotherapy in patients with axillary node-positive breast cancer by combining radioactive iodine seed localization in the axilla with the sentinel node procedure (RISAS): a dutch prospective multicenter validation study. Clin Breast Cancer.

[13] Straver ME, Loo CE, Alderliesten T, Rutgers EJ, Vrancken Peeters MT (2010). Marking the axilla with radioactive iodine seeds (MARI procedure) may reduce the need for axillary dissection after neoadjuvant chemotherapy for breast cancer. Br J Surg.

[14] Breast Cancer Guideline, NABON 2012. Dutch Guideline, Version 2.0, 2012. https://www.oncoline.nl/uploaded/docs/mammacarcinoom/Dutch%20Breast%20Cancer%20Guideline%202012.pdf.

[15] Koolen BB, Valdés Olmos RA, Elkhuizen PH, Vogel WV, Vrancken Peeters MJ, Rodenhuis S (2012). Locoregional lymph node involvement on 18F-FDG PET/CT in breast cancer patients scheduled for neoadjuvant chemotherapy. Breast Cancer Res Treat.

[16] Koolen BB, Valdés Olmos RA, Vogel WV, Vrancken Peeters MJ, Rodenhuis S, Rutgers EJ (2013). Pre-chemotherapy 18F-FDG PET/CT upstages nodal stage in stage II–III breast cancer patients treated with neoadjuvant chemotherapy. Breast Cancer Res Treat.

[17] Aukema TS, Straver ME, Peeters MJ, Russell NS, Gilhuijs KG, Vogel WV (2010). Detection of extra-axillary lymph node involvement with FDG PET/CT in patients with stage II–III breast cancer. Eur J Cancer.

[18] Teixeira SC, Koolen BB, Vogel WV, Wesseling J, Stokkel MP, Vrancken Peeters MJ (2016). Additional prone 18F-FDG PET/CT acquisition to improve the visualization of the primary tumor and regional lymph node metastases in stage II/III breast cancer. Clin Nucl Med.

[19] Williams JM, Rani SD, Li X, Arlinghaus LR, Lee TC, MacDonald LR (2015). Comparison of prone versus supine 18F-FDG-PET of locally advanced breast cancer: phantom and preliminary clinical studies. Med Phys.

[20] Koolen BB, Vrancken Peeters MJ, Aukema TS, Vogel WV, Oldenburg HS, van der Hage JA (2012). 18F-FDG PET/CT as a staging procedure in primary stage II and III breast cancer: comparison with conventional imaging techniques. Breast Cancer Res Treat.

[21] Kaida H, Ishibashi M, Fuji T, Kurata S, Uchida M, Baba K (2008). Improved breast cancer detection of prone breast fluorodeoxyglucose-PET in 118 patients. Nucl Med Comm.

[22] Fuster D, Duch J, Paredes P, Velasco M, Muñoz M, Santamaría G (2008). Preoperative staging of large primary breast cancer with [18F]fluorodeoxyglucose positron emission tomography/computed tomography compared with conventional imaging procedures. J Clin Oncol.

[23] van der Hoeven JJ, Hoekstra OS, Comans EF, Pijpers R, Boom RP, van Geldere D (2002). Determinants of diagnostic performance of [F-18]fluorodeoxyglucose positron emission tomography for axillary staging in breast cancer. Ann Surg.

[24] Avril N, Rosé CA, Schelling M, Dose J, Kuhn W, Bense S (2000). Breast imaging with positron emission tomography and fluorine-18 fluorodeoxyglucose: use and limitations. J Clin Oncol.

[25] Yutani K, Tatsumi M, Uehara T, Nishimura T (1999). Effect of patients' being prone during FDG PET for the diagnosis of breast cancer. AJR Am J Roentgenol.

[26] Khalkhali I, Mena I, Diggles L (1994). Review of imaging techniques for the diagnosis of breast cancer: a new role of prone scintimammography using technetium-99m sestamibi. Eur J Nucl Med.

[27] Abramson RG, Lambert KF, Jones-Jackson LB, Arlinghaus LR, Williams J, Abramson VG (2015). Prone versus supine breast FDG-PET/CT for assessing locoregional disease distribution in locally advanced breast cancer. Acad Radiol.

[28] Koolen BB, Vogel WV, Vrancken Peeters MJ, Loo CE, Rutgers EJ, Valdés Olmos RA. Molecular Imaging in Breast Cancer: From Whole-Body PET/CT to Dedicated Breast PET. J Oncol. 2012;2012:438647. 10.1155/2012/438647 PMC3400419 22848217

[29] van der Noordaa MEM, van Duijnhoven FH, Loo CE, van Werkhoven E, van de Vijver KK, Wiersma T (2018). Identifying pathologic complete response of the breast after neoadjuvant systemic therapy with ultrasound guided biopsy to eventually omit surgery: study design and feasibility of the MICRA trial (Minimally Invasive Complete Response Assessment). Breast.

[30] Donker M, Drukker CA, Valdés Olmos RA, Rutgers EJ, Loo CE, Sonke GS (2013). Guiding breast-conserving surgery in patients after neoadjuvant systemic therapy for breast cancer: a comparison of radioactive seed localization with the ROLL technique. Ann Surg Oncol.

[31] Alderliesten T, Loo CE, Pengel KE, Rutgers EJ, Gilhuijs KG, Vrancken Peeters MJ (2011). Radioactive seed localization of breast lesions: an adequate localization method without seed migration. Breast J.

[32] Mann RM, Cho N, Moy L (2019). Breast MRI: state of the art. Radiology.

[33] Sawicki LM, Grueneisen J, Schaarschmidt BM, Buchbender C, Nagarajah J, Umutlu L (2016). Evaluation of ^18^F-FDG PET/MRI, ^18^F-FDG PET/CT, MRI, and CT in whole-body staging of recurrent breast cancer. Eur J Radiol.

[34] Plaza MJ, Collado-Mesa F, Bokhoor J, Alperin N, Yepes MM (2014). Diagnostic performance of CT attenuation values of focal 18F-FDG avid breast lesions detected on whole-body PET-CT in postoperative breast cancer patients. Breast J.

[35] van Loevezijn AA, van der Noordaa MEM, van Duijnhoven FH, Groen EJ, Stokkel MPM, Loo CE, Elkhuizen P, Russell NS, Vrancken Peeters MTFD (2020). Tailored axillary treatment after neoadjuvant systemic therapy inclinically node-positive breast cancer patients is safe: 3-yearfollow-up of the MARI protocol. Eur J Cancer.

