# Supplementary material for: [18F]FDG-PET/CT in prone compared to supine position for optimal axillary staging and treatment in clinically node-positive breast cancer patients with neoadjuvant systemic therapy
Source: EJNMMI Res. 2021 Aug 21;11:78. doi: 10.1186/s13550-021-00824-4 (PMC8380204; doi:10.1186/s13550-021-00824-4)
Supplement: Supplementary file 1 — Additional file 1.Table S1. Number of patients categorized in the same axillary staging groups with FDG-PET/CT in supine and prone position by both observers. [file 13550_2021_824_MOESM1_ESM.pdf]

SUPPLEMENTARY APPENDIX

van Loevezijn A.A., Stokkel M.P.M., Donswijk L.M., van Werkhoven E.D., van der Noordaa M.E.M, van Duijnhoven F.H., Vrancken Peeters M.T.F.D (2021). 18F-FDG PET/CT in prone compared to supine position for optimal axillary staging in breast cancer patients undergoing tailored axillary treatment after neoadjuvant systemic therapy according to the MARI-protocol. Breast Cancer Research and Treatment.

Corresponding author:
Marie-Jeanne T.F.D. Vrancken Peeters, Professor of Surgical Oncology
Netherlands Cancer Institute - Antoni van Leeuwenhoek
E-mail: m.vrancken@nki.nl

Table S1. Number of patients categorized in the same axillary staging groups with FDG-PET/CT in supine and prone position by both observers.

	**Observer 1 Supine / Prone**									
	N<4 / N<4		N<4 / N4+		N4+ / N<4		N4+ / N4+		Total	
**Observer 2 Supine / Prone**										
N<4 / N<4	**92**	(60.1%)	1	(0.7%)	0	-	0	-	93	(60.8%)
N<4 / N4+	12	(7.8%)	**0**	-	1	(0.7%)	1	(0.7%)	14	(9.2%)
N4+ / N<4	2	(1.3%)	0	-	**1**	(0.7%)	0	-	3	(1.9%)
N4+ / N4+	5	(3.3%)	3	(1.9%)	3	(1.9%)	**32**	(20.9%)	43	(28.1%)
Total	111	(72.5%)	4	(2.6%)	5	(3.3%)	33	(21.6%)	153	(100%)

Data are *N*, (%). *N<4* less than four FDG-positive axillary lymph nodes; *N4+* more than four FDG-positive axillary nodes.
